# Dexmedetomidine combined with sufentanil and dezocine-based patient-controlled intravenous analgesia increases female patients’ global satisfaction degree after thoracoscopic surgery

**DOI:** 10.1186/s13019-021-01472-4

**Published:** 2021-04-21

**Authors:** Qiongzhen Li, Haixia Yao, Meiying Xu, Jingxiang Wu

**Affiliations:** Department of Anesthesiology of Shanghai Chest Hospital, Shanghai Jiaotong University, No. 241 Huaihai Rd. West, Shanghai, 200025 China

**Keywords:** Satisfaction degree, Sufentanil, Dexmedetomidine, Dezocine, Combination, Thoracoscopic, Patient-Controlled Intravenous Analgesia.

## Abstract

**Background:**

There are no studies on the use of dexmedetomidine combined with sufentanil and dezocine-based patient-controlled intravenous analgesia (PCIA) in females undergoing thoracic surgery. We postulate that introducing dexmedetomidine to a combination of dezocine-based PCA drugs and sufentanil will increase female patients’ global satisfaction degree.

**Methods:**

One hundred fifty-two female patients with physical classification type I or II according to the American Society of Anesthesiologists undergoing thoracoscopic surgery were arbitrarily classified into two categories, either receiving sufentanil and dezocine-based PCIA (group C) or incorporating dexmedetomidine with sufentanil and dezocine-based PCIA (group D). The patients’ global satisfaction degree, postoperative nausea and vomiting (PONV), PCA bolus, rescue analgesia requirements, drug-related adverse effects, rest and coughing visual analogue scale (VAS) ratings, and Ramsay sedation scores (RSS) were measured at 6, 12, 24, 36 and 48 h after surgery.

**Results:**

Compared with the C group, the patient satisfaction degree was significantly higher; pain scores at rest and coughing were significantly different at 6, 12, 24, 36 and 48 h postoperatively; less rescue analgesia and PCA bolus were required; and a lower incidence of PONV was found in the D group. There were non-significant trends for the sedation scores and drug-related adverse effects in both groups.

**Conclusions:**

Dexmedetomidine combined with sufentanil and dezocine increased female patients’ global satisfaction degree after thoracoscopic surgery. This effect could be linked to the improvement in postoperative analgesia and reduction in postoperative nausea and vomiting; the combined treatment did not increase drug-related adverse effects in female patients.

**Trial registration:**

Chinese Clinical Trial Registry number, ChiCTR2000030429. Registered on March 1, 2020.

## Introduction

With the emergence of enhanced recovery after surgery (ERAS), adequate pain control has been reported to enhance surgical results leading to decreased morbidity, hospitalisation and convalescence, and it is generally accepted that adequate pain management is a requirement for early postoperative rehabilitation [1]. Patients undergoing thoracic surgery experience serious pain with major effects from respiratory movements during the postoperative period [2, 3]. The most widely used analgesic approach continues to be patient-controlled intravenous analgesia (PCIA). Opioids are analgesics that are frequently used for PCIA. While increased dosages of opioid pharmaceutical products may improve postoperative pain, undesirable drug-related consequences, including pruritus, vomiting, nausea and respiratory distress, often occur, especially for female patients [4, 5].

Dezocine is a serotonin-norepinephrine reuptake inhibitor and functions as a partial μ-receptor agonist and κ-receptor antagonist [6]. Small doses of dezocine combined with morphine increase the effectiveness of postoperative analgesia for thoracotomy [7]. Dexmedetomidine is a highly selective α_2_ adrenergic receptor agonist that hypnotic, sedative, analgesic and anxiolytic actions and does not cause respiratory depression. Dexmedetomidine can also increase the analgesic efficacy of opioids [8–13]. However, the mechanisms of the effect of dexmedetomidine when combined with opioids remain unclear.

In this randomised, placebo-controlled, double-blind study, we hypothesised that the addition of dexmedetomidine to sufentanil and dezocine-based PCA drug mixtures would improve female patient satisfaction. The secondary goal was to assess the analgesic potency and undesirable outcomes of dexmedetomidine 48 h after thoracoscopic surgery.

## Methods

### Patients and data collection

A total of 152 female patients who were 30–60 years of age, were American Society of Anesthesiologists (ASA) class I-II, and presented for three-trocar video-assisted thoracoscopic surgery from February 24, 2020, to April 2, 2020, were enrolled. The Shanghai Jiaotong University Shanghai Chest Hospital (KS1865) Academic Review Board authorised the research procedure for surgeries involving general anaesthesia. We registered this research at Chictr.org (ChiCTR2000030429). Written informed consent was obtained from all participants.

### Inclusion criteria

The female patients were selected as follows: 1) capacity to comprehend Chinese verbal and written, 2) American Society of Anesthesiologists physical status I to II, 3) age 30 to 60 years, 4) scheduled for three-trocar VATS under general anesthesia, and 5) received 48 h continual PCIA after surgery.

### Exclusion criteria

The exclusion criteria were as follows: 1) age under 30 or over 60 years, 2) refusal to participate, 3) use of psychiatric medications and alcohol abuse, 4) history of cardiovascular disease, 5) acute or chronic liver or kidney disease, 6) cognitive impairment, and 7) pregnancy or lactation.

### Randomisation

Patients were randomised with a 1:1 equal allocation ratio to sufentanil and dezocine-based PCIA (group C) or combined dexmedetomidine with sufentanil and dezocine-based PCIA (group D). A clinical trial statistician provided randomisation by using random number table. After written informed consent, the anaesthetist began randomisation of the patients using the online database. Later, in the procedure room, the randomised treatment was started.

### Blinding

The patients and the research team were not informed as to whether the patients were assigned to the sufentanil and dezocine-based PCIA group (group C) or the combined dexmedetomidine with sufentanil and dezocine-based PCIA group (group D) until the end of the study. Only the clinical trial statistician and dispensing nurse know the group allocation. The blinding could be disrupted in case of emergency if the patients’ health or safety were at risk.

### Methods of Anaesthesia

According to the clinical research center protocol, when the patient arriveed at the anaesthesia preparation room, a peripheral vein was opened using a catheter, and right internal jugular central venous catheterisation was performed. When the participants were released to the operating room, in addition to a normal evaluation via pulse oximetry, non-invasive blood pressure (NIBP) and electrocardiography (ECG), the arterial pressure was assessed using a radial artery catheter in all patients. During surgery, surface electrodes for the bi-spectral index were placed on the front of the patients; BIS was maintained between 40 and 50. For both groups, a standardised anaesthetic method was used. Induction and upkeep of anaesthesia were the responsibility of the participating anaesthesiologists who were blinded to group assignments. Ten minutes before the induction of anaesthesia, 0.5 μg/kg dexmedetomidine (DEX) was in group D or normal saline in group C was added to a 20-ml syringe for administration over 10 min. Anaesthesia was subsequently induced at an effect-site concentration (Ce) of 4 μg/ml with sufentanil (0.5 μg/kg), cisatracurium (0.2 mg/kg), 50 mg flurbiprofen, and 2% propofol using a target-controlled infusion (TCI). The patients were intubated with a DLT in the lateral position, which required no operation. The correct position was confirmed with the use of a fibreoptic bronchoscope (FOB). Throughout the initial two-lung ventilation (TLV) and one-lung ventilation (OLV) cycles, the tidal volume was 7 ml/kg, which maintained PetCO_2_ at 35–45 mmHg (I/E = 1:2, f = 12). Anaesthesia was maintained using cisatracurium (0.12 mg/kg/h), remifentanil (0.1–0.3 μg/kg/min) and 2% propofol, with Ce at 2–3 μg/ml titrated to maintain BIS between 40 and 50; the mean arterial blood pressures (MAPs) and heart rates (HRs) were 20% less than the baseline values. The nasopharyngeal temperature was maintained at ≥36.5 °C. FOB was used to validate the correct DLT location after the patient was placed in a lateral decubitus position once more. Using 100% oxygen, anaesthesia induction and OLV were introduced and sustained. At the end of the operation, the inspired concentration of oxygen was decreased by 50% and the positive end expiratory pressure was increased to 5 cmH_2_O after the lung recruitment manoeuvre. At the end of surgery, intravenous infusion of all anaesthetics were stopped, a palonosetron hydrochloride injection (0.25 mg for a single injection) was given to prevent nausea and vomiting, and an electronic infusion pump (FSQ-11 PCA; Inc., JiangSu AIPENG, ED, China) for PCIA was connected. After surgery, patients were transferred to a post anaesthesia care unit (PACU). When fully awake, the patients were transferred to a regular ward and monitored during the whole study period.

### Postoperative PCIA strategy

In the PCIA protocol, the sufentanil and dezocine levels were determined based on body weight in the study timeframe. As per the study design, the PCIA base regimen was 1.5 μg/kg sufentanil and 0.3 mg/kg dezocine diluted to 100 ml with 0.9% normal saline. In the D group, in addition to the sufentanil and dezocine, 3.0 μg/kg dexmedetomidine was added to the PCIA pump. The PCIA was set to a background infusion rate of 2 ml/h, lock-out interval of 15 min and 0.5 ml bolus on demand. All study patients received a 0.5 ml i.v. of PCIA solution while connected to a PCIA pump. The PCIA was set to a continuous background infusion of 0.03 μg/kg/h sufentanil with a bolus of 0.02 μg/kg sufentanil and allowed a continuous background infusion of 0.06 μg/kg/h dexmedetomidine with a bolus of 0.03 μg/kg dexmedetomidine. The PCA was used continuously for the first 48 h postoperatively. If patients complained of extreme discomfort, higher than a VAS level of 5, Once released to the general ward, the physicians advised them to push the PCA button, and if comfort was not achieved, then the nurse would administer a rescue analgesic of 50 mg flurbiprofen intravenously during the 48 h study period.

### Measurements

#### Primary outcome

The primary endpoint measures was the satisfaction degree in the two groups within 48 h after surgery. The satisfaction degree scores were from 1 to 4 (4 = very satisfied, 3 = satisfied, 2 = moderately satisfied, and 1 = not satisfied).

#### Secondary outcomes

The secondary endpoint measures were postoperative nausea and vomiting (PONV), PCA bolus, requirement for rescue analgesia, drug-related adverse effects, the visual analogue scale (VAS) pain scores at rest and while coughing, and the Ramsay sedation score (RSS) at 6, 12, 24, 36 and 48 h postoperatively. The operative times and the incidences of hypotension, atrial fibrillation and sinus bradycardia.

The nausea and vomiting (PONV) ratings were on a 4-point scale (1 = without nausea and vomiting, 2 = nausea without vomiting, 3 = less than twice vomiting, 4 = extreme vomiting more than twice), and the sedation score was on a 6-point sedation scale (1 = fully awake; 2 = cooperative, calm; 3 = response only to verbal commands; 4 = vigorous response to light stimulation while asleep; 5 = sleeping without light reaction; 6 = unarousable) [14]. The degree of resting and coughing pain from the operation was assessed at 6, 12, 24, 36 and 48 h. A numerical rating scale from 0 to 10 (0 indicates no pain at all and 10 reflects the worst pain known) was used to measure pain severity [8].

The 48-h analgesia pump PCA bolus and rescue analgesia were recorded, and the occurrences of nausea and vomiting, pruritus, hypotension (MAP < 60 mmHg), sinus bradycardia (HR < 60 beats/min), excessive sedation (RSS ≥3), hypoxaemia (SpO_2_ < 93%), and respiratory depression (respiratory rate < 8 bpm) were recorded for 48 h postoperatively.

### Statistical analysis

Quantitative variables are reported as the mean ± standard deviation. Categorical data are defined in terms of frequencies and fractions. The statistical study was carried out using SPSS version 22 (SPSS, Chicago, IL). The χ^2^ test or Fisher’s exact test was performed to compare all categorical variables. Continuous variables were tested with the non-parametric Mann-Whitney U test or t-test depending on the distribution of the data. Two-sided *p*-values < 0.05 were considered as statistically significant. The sample size was estimated based on an anticipated 20% drop in the satisfaction degree ratio for 48 h post-surgery. For an 80% power (α = 0.05, β = 0.2), the sample size needed for each group was estimated to be 69, with a dropout rate of 10%. There were 76 patients per group for eligibility.

## Results

**Fig. 1:**
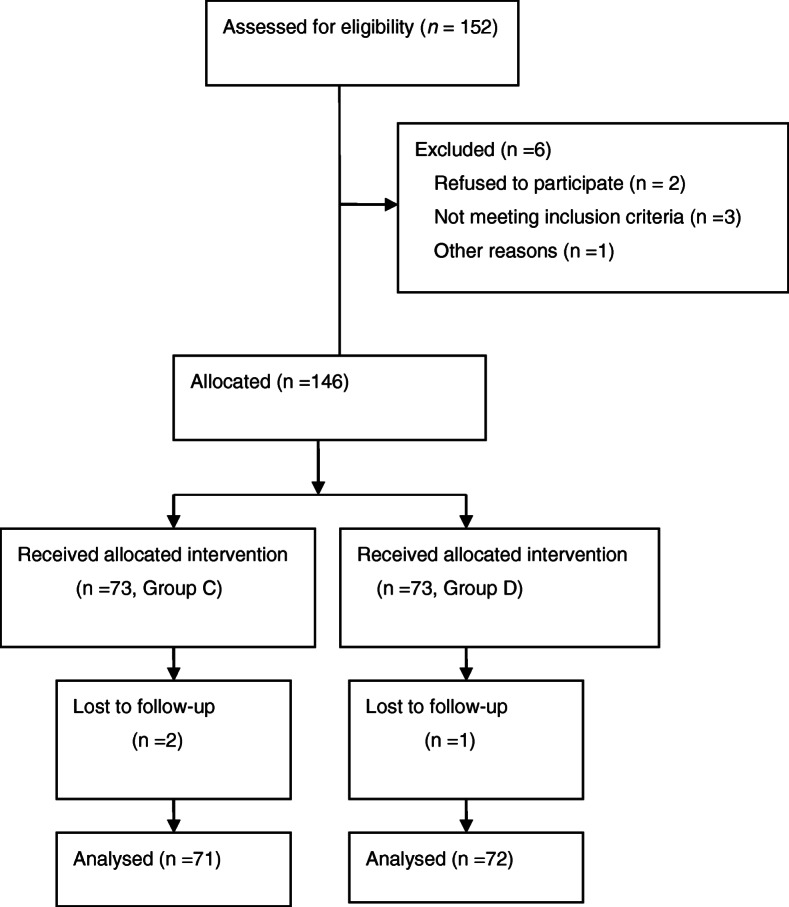
CONSORT flow diagram

A total of 152 female patients were included in this study, but nine were omitted. Eventually, data from 143 patients were entered for final examination (Fig. 1). There were no major variations between the demographic information of the groups (Table 1), namely, height, age, weight, ASA grade, operating period, intraoperative sufentanil and remifentanil intake, or right/left lobectomy resection (*P* > 0.05, Table 1).

**Table 1:** Patient characteristics

Variable	Group C (*n* = 71)	Group D (*n* = 72)	*P* values
Age (years)	51.7 ± 7.9	50.3 ± 6.90	0.272
Weight (kg)	58.3 ± 7.1	57.9 ± 7.3	0.755
Height (cm)	160.1 ± 5.5	159.3 ± 5.9	0.413
ASA class I/II(n)	34/37	39/33	0.505
Time in surgery (min)	122.8 ± 7.6	120.4 ± 7.2	0.063
Intraoperative sufentanil consumption (μg)	29.3 ± 3.9	29.2 ± 3.6	0.943
Intraoperative remifentanil consumption (μg)	1406.8 ± 172.6	1391.0 ± 174.6	0.588
Right/Left lobectomy resection (n)	31/40	35/37	0.616

Data are expressed as the means ± SD or number

The patients in group D had a slightly greater degree of satisfaction than the patients in group C (*p* < 0.05, Table 2). Table 3 displays the rescue analgesia and the PCA pump bolus. Post-operative rescue analgesia showed major differences between the groups. The D group required less rescue analgesia within 48 h postoperatively compared with the C group (*P* < 0.05, Table 3). In the D group (*P* < 0.05, Table 3), the amounts of the cumulative boluses, actual PCA boluses, and unsuccessful boluses within 48 h were smaller than those in the C group.

**Table 2:** Satisfaction degree within 48 h

Variable	Group C (*n* = 71)	Group D (*n* = 72)	*P* values
Not satisfied	14 (19.7%)	4 (5.5%)*	0.012
Less satisfied	26 (36.6%)	11 (15.3%)*	0.004
Satisfied	29 (40.9%)	44 (61.1%)*	0.019
Very satisfied	2 (2.8%)	13 (18.1%)*	0.005

Values are numbers (%).

*Statistically significant (*P* < 0.05)

**Table 3:** Postoperative rescue analgesia and the number of PCA boluses

Variable	Group C (*n* = 71)	Group D (*n* = 72)	*P* values
Rescue analgesia			
No	50 (70.4%)	66 (91.7%)*	0.001
Once	12 (16.9%)	4 (5.6%)*	0.036
Twice	9 (12.7%)	2 (2.7%)*	0.031
PCA bolus			
Total	14.3 ± 3.3	10.5 ± 3.9*	< 0.001
Actual	11.8 ± 3.2	9.0 ± 3.5*	< 0.001
Ineffective	2.5 ± 1.6	1.5 ± 0.9*	< 0.001

Values are means ± SD or numbers (%)

*Statistically significant (*P* < 0.05)

Compared with the C group, the Ramsay score had no significant differences at 6, 12, 24, 36 and 48 h postoperatively (*P* > 0.05, Table 4), and the VAS scores at rest and while coughing were lower at 6, 12, 24, 36 and 48 h postoperatively in the D group (*P* < 0.05, Table 4).

**Table 4:** VAS and Ramsay scores within 48 h postoperatively

Variable	Time	Group C (*n* = 71)	Group D (*n* = 72)	*P* values
Rest VAS	6	2.0 ± 0.9	1.6 ± 0.9*	0.010
	12	2.0 ± 1.0	1.7 ± 0.9*	0.025
	24	2.1 ± 1.2	1.7 ± 0.9*	0.023
	36	2.0 ± 1.5	1.5 ± 1.1*	0.030
	48	2.0 ± 1.4	1.5 ± 0.8*	0.005
Cough VAS	6	3.0 ± 0.9	2.5 ± 0.6*	< 0.001
	12	3.1 ± 1.2	2.3 ± 1.1*	< 0.001
	24	3.0 ± 1.0	2.7 ± 0.9*	0.041
	36	3.0 ± 1.4	2.5 ± 1.1*	0.032
	48	3.0 ± 1.3	2.3 ± 0.8*	< 0.001
Ramsay	6	2.2 ± 0.5	2.3 ± 0.5	0.060
	12	2.0 ± 0.2	2.1 ± 0.3	0.124
	24	2.1 ± 0.4	2.2 ± 0.4	0.077
	36	2.0 ± 0.2	2.1 ± 0.3	0.097
	48	2.0 ± 0.3	2.1 ± 0.3	0.058

Values are means ± SD

*Statistically significant (*P* < 0.05)

Compared with the C group, the incidence of nausea and vomiting and rescue antiemetics within 48 h postoperatively were significantly lower in the D group (*p* < 0.05, Table 5). We found no respiratory depression during 48 h after surgery (*P* > 0.05, Table 6).

**Table 5:** Incidence of postoperative nausea and vomiting and rescue antiemetics within 48 h after surgery

Variable	Group C (*n* = 71)	Group D (*n* = 72)	*P* values
Without nausea and vomiting	28 (39.4%)	55 (76.4%)*	< 0.001
Nausea without vomiting	21 (29.6%)	10 (13.9%)*	0.026
Vomiting≤2 times	10 (14.1%)	3 (4.1%)*	0.046
Vomiting> 2 times	12 (16.9%)	4 (5.6%)*	0.036
Rescue antiemetics	16 (22.5%)	7 (9.7%)*	0.043

Values are numbers (%).

*Statistically significant (*P* < 0.05)

**Table 6:** Adverse effects of postoperative analgesia

Variable	Group C (*n* = 71)	Group D (*n* = 72)	*P* values
Pruritus	5 (7.0%)	4 (5.6%)	0.745
Hypertension	3 (4.2%)	5 (6.9%)	0.719
Hypotension	1 (1.4%)	2 (2.8%)	1.000
Sinus bradycardia	1 (1.4%)	3 (4.2%)	0.620
Excessive sedation	3 (4.2%)	5 (6.9%)	0.719
Hypoxaemia	2 (2.8%)	1 (1.4%)	0.620
Atrial fibrillation	0	0	–
Respiratory depression	0	0	–

Values are numbers (%)

*Statistically significant (*P* < 0.05). hypertension (MAP > 90 mmHg); hypotension (MAP < 60 mmHg); sinus bradycardia (HR < 60 beats/min); excessive sedation (RSS ≥3); hypoxaemia (SpO2 < 93%); respiratory depression (respiratory rate < 8 bpm)

## Discussion

In this randomised controlled study, dexmedetomidine introduced to a sufentanil and dezocine-based PCA drug blend increased female patients’ postoperative global satisfaction degree, reduced the severity and frequency of vomiting and nausea and the requirement for rescue analgesia, minimised the consumption of analgesic, and decreased postoperative pain scores without increasing the incidence of clinically relevant hypertension, hypotension, excessive sedation, sinus bradycardia, hypoxaemia or respiratory depression during the first 48 h after thoracoscopy.

Opioids, such as sufentanil and morphine, are commonly used with postoperative analgesia and various forms of treatment. Nevertheless, opioid-related adverse effects, such as addiction, respiratory depression, diarrhoea, constipation, pruritus and sedation, compel us to seek novel medications that are suitable as postoperative analgesics to minimise the use of opioids and reduce their negative impacts and have suitable postoperative analgesics. Multimodal analgesia of medications has proven successful [15].

Dezocine is a combined agonist and antagonist of combined opioid receptors, and an increasing amount of research has demonstrated that dezocine use with opioids may decrease opioid intake and the adverse effects correlated with opioids. For instance, Wu et al [7] reported that low dezocine levels could improve postoperative analgesia, nausea and pruritus following thoracotomy. Furthermore, Yu et al [16] showed that dezocine provides a substantial postoperative antihyperalgesic and analgesic impact for up to 48 h on patients receiving elective open gastrectomy. Dexmedetomidine is an agonist of the highly selective receptor α_2_. Dexmedetomidine is quite appropriate for use as a part of multimodal analgesia because of its analgesic, sedative, hypnotic, and anti-sympathetic effects. Patient-controlled analgesia paired with dexmedetomidine is used to reduce the side effects associated with opioids [17, 18].

Patients experience serious pain after thoracoscopic surgery. Effective postoperative analgesia can reduce pain scores, the PCA bolus, and the need for rescue analgesia and increase the overall level of comfort of from analgesic in female patients in the 48 h after surgery. Such findings showed that introducing dexmedetomidine to sufentanil and PCA based on dezocine could improve the analgesic effects, providing female patients with a better analgesic experience.

PONV is undeniably quite stressful, and even a slight episode will dramatically decrease the degree of patient satisfaction, postpone hospital discharge and increase the utilisation of medical services, particularly for high-risk female patients [19]. This research found that PONV occurrence could be decreased by the addition of dexmedetomidine to sufentanil and dezocine-based PCA. Previous research has indicated that perioperative use of dexmedetomidine could minimise the occurrence of nausea and vomiting [20, 21]. This anti-nausea property may be explained by the direct anti-nausea and anti-vomiting functions of α_2_ receptor agonists. Furthermore, PONV may be decreased by the anti-sympathetic qualities of dexmedetomidine.

The research presented herein revealed no substantial difference in the degree of sedation between the two groups. Dexmedetomidine has sedative qualities and acts on the subcortical system to generate sedation and enhance the quality of postoperative sleep [22–24]. Hence, dexmedetomidine is known to offer sedation without a respiratory disturbance, and the Ramsay sedation scores (RSS) did not indicate unnecessary sedation either. Dexmedetomidine sedation is close to normal sleep, suggesting that the formulation of 0.06 μg/kg/h dexmedetomidine used in our research procedure did not produce severe sedation of therapeutic importance but may have improved the level of satisfaction of the female patients.

In this clinical trial study, there were some limitations. Firstly, we assessed the degree of satisfaction using the patients’ rating on a scale asked by an examiner; this method was subjective. Secondly, because of the time constraints, the sample size was not large enough. Furthermore, this is only a single center study; and there is a need for a multi-center study with a large sample size. In addition, discrepancies in hospitalisation length and medical billing were not assessed and will be examined in future research. A critical limitation is the exclusion of men which limits the generalization of the study findings. Finally, a long-term analysis on the improvement in degree of satisfaction many months later is needed, as research has demonstrated that there may be a correlation between acute postoperative pain and the likelihood of developing chronic pain [25].

In conclusion, combining dexmedetomidine with a sufentanil and dezocine-based PCA drug mixture could improve female patients satisfaction degree in the first 48 h of after thoracoscopic surgery, providing good analgesic efficacy, less postoperative nausea and vomiting, and no increase in postoperative adverse events.

## Conclusions

Dexmedetomidine combined with sufentanil and dezocine-based increased female patients’ global satisfaction degree after thoracoscopic surgery. This effect could be linked to the improvement in postoperative analgesia and reduction in postoperative nausea and vomiting, and did not increase drug-related adverse effects in female patients.

## Data Availability

The authors do not wish to share their data because the patients who participated in this study did not agree to share their individual data.

## Abbreviations

ASA: American Society of Anesthesiologists
BIS: Bispectral index
OLV: One-lung ventilation
TLV: Two-lung ventilation
DLT: Double-lumen tube
HR: Heart rate
ECG: Electrocardiography
MBP: Mean blood pressure
NIBP: Non-invasive blood pressure
SpO_2_: Pulse oxygen saturation
TCI: Target-controlled infusion
Ce: Effect-site concentration
I:E: Inspiration/expiration
FOB: Fibreoptic bronchoscopy
VATS: Video-assisted thoracoscopic surgery
PEEP: Positive end expiratory pressure
PACU: Post-anaesthetic care unit
DEX: Dexmedetomidine
SD: Standard deviation
PCIA: Patient-controlled intravenous analgesia
PONV: Postoperative nausea and vomiting
VAS: Visual analogue scale cores
RSS: Ramsay sedation score
ERAS: Enhanced recovery after surgery
